# Collaborative teaching among ultrasonographers, anesthesiologists, and vascular surgeons: its unique role in specialty training for vascular surgeons in developing countries

**DOI:** 10.3389/fmed.2024.1446910

**Published:** 2024-10-11

**Authors:** Peng Zhang, Mao Zhang, Qingfeng Zhang, Wei Hu

**Affiliations:** ^1^Department of Anesthesia, Sichuan Provincial People’s Hospital, University of Electronic Science and Technology of China, Chengdu, China; ^2^Department of Vascular Surgery, Sichuan Provincial People's Hospital, University of Electronic Science and Technology of China, Chengdu, China; ^3^Department of Ultrasonic, Sichuan Provincial People's Hospital, University of Electronic Science and Technology of China, Chengdu, China

**Keywords:** collaborative teaching, training of vascular surgeons, developing countries, ultrasound technology, anesthesia skills, interdisciplinary cooperation

## Abstract

This study explored the unique role of collaborative teaching among ultrasonographers, anesthesiologists, and vascular surgeons in the training of vascular surgeons in developing countries. Using qualitative research methods, data were collected through in-depth interviews and observations to analyze the improvement in ultrasound operational skills and basic anesthesia skills among trainees, as well as their satisfaction with collaborative teaching. The results indicate that collaborative teaching significantly enhances trainees’ professional skills and interdisciplinary collaboration abilities, gaining widespread recognition from trainees. This teaching model provides trainees with a comprehensive learning experience through interdisciplinary cooperation, hands-on practice, contributing to the cultivation of vascular surgeons with comprehensive qualities and professional competencies. This study offers new ideas and methods for the training of vascular surgeons in developing countries, holding significant implications for the development of medical education.

## 1 Introduction

With the rapid development of global medical technology, the field of vascular surgery has undergone unprecedented changes. From initially relying primarily on hemostasis and basic open surgery methods to the widespread application of minimally invasive techniques such as in endovascular surgery, the treatment modalities in vascular surgery have achieved a qualitative leap (1). Simultaneously, the application of 3D printing technology in vascular surgery has provided new solutions for surgical planning and simulations (2). Especially in the 21st century, these changes have become even more pronounced, not only improving the precision and safety of surgeries but also bringing better treatment outcomes and quality of life to patients. However, the widespread application of these advanced technologies and equipment also poses a series of challenges, particularly for developing countries with relatively limited medical resources.

In developing countries, due to insufficient hardware facilities and constraints on medical costs, many hospitals may not be able to equip themselves with expensive Digital Subtraction Angiography (DSA) operating rooms or other high-end medical equipment. This results in doctors facing different conditions and limitations when dealing with the same medical issues. Therefore, how to improve the clinical skills and professional competence of vascular surgeons under limited resources has become an important issue before us.

Ultrasound technology, as a non-invasive and relatively low-cost diagnostic and therapeutic tool, holds unique advantages in the field of vascular surgery. It plays a crucial role not only in the diagnosis of vascular surgical diseases but also as an essential guidance tool during surgery, helping doctors accurately locate vascular lesions, assess blood flow conditions, and guide surgical operations (3, 4). Additionally, ultrasound holds irreplaceable value in post-surgical monitoring. Through postoperative ultrasound examinations, doctors can conveniently and promptly evaluate surgical outcomes, observe the healing of surgical sites, and detect and manage postoperative complications (5).

In vascular surgery, nerve block anesthesia is a commonly used anesthesia method. Examples include femoral nerve block in lower extremity vascular surgery and brachial plexus block in upper extremity arterial thrombectomy (6). Ultrasound-guided nerve block anesthesia can accurately locate the target nerve and inject local anesthetics around the nerve under direct vision, thereby effectively blocking nerve impulse conduction and achieving anesthesia. Compared with traditional nerve block methods, ultrasound-guided nerve block anesthesia has higher success rates and safety, and can reduce the dosage of local anesthetics and the risk of nerve damage. However, vascular surgeons often lack the skills for simple nerve block anesthesia and often require the assistance of anesthesiologists. Mastering these skills, however, would enable surgeons to perform certain simple surgeries without the presence of anesthesiologists, thereby reducing staffing requirements and medical expenses for patients. This is particularly crucial for hospitals in low- and middle-income countries with limited medical resources.

Despite the widespread application of ultrasound technology in vascular surgery, many vascular surgeons, especially those in primary hospitals, still lack proficiency in ultrasound operation skills and basic anesthesia skills based on ultrasound (7). This limitation, to a certain extent, constrains their ability to independently handle cases in clinical practice. Therefore, exploring an effective training model to enhance the ultrasound operation skills and basic anesthesia skills of vascular surgeons has become an urgent issue.

Against this backdrop, we propose this collaborative teaching research. Collaborative teaching, a method where professionals from different disciplines work together to educate trainees, has gained recognition for its potential to enhance healthcare education and improve patient care quality (8). This method leverages the diverse expertise of various professionals to provide a more comprehensive and practical learning experience for trainees. In the context of vascular surgery training, especially in developing countries, the integration of ultrasonographers, anesthesiologists, and vascular surgeons through collaborative teaching can address critical gaps in resources and expertise.

In many developing low- and middle-income countries (LMICs), healthcare systems often face significant challenges, including limited resources and a shortage of specialized medical professionals (9). The World Health Organization (WHO) has highlighted the importance of task shifting and collaborative approaches in these settings to maximize the use of available resources and enhance the quality of care (10, 11). Task shifting involves delegating tasks traditionally performed by specialized professionals to those with less training but adequate skills, thereby optimizing the workforce’s efficiency (12).

The aim of this study is to evaluate the effectiveness of collaborative teaching among ultrasonographers, anesthesiologists, and vascular surgeons in the specialty training of vascular surgeons in developing countries. Specifically, we sought to determine the impact of this teaching method on trainees’ skills in ultrasonography and anesthesia, as well as their overall satisfaction with the training program.

Objectives of the study include:

1. Assessing the improvement in ultrasonography skills among vascular surgery trainees.
2. Evaluating the enhancement of basic anesthesia skills in the trainees.
3. Measuring the trainees’ satisfaction with the collaborative teaching approach.
4. Identifying the benefits and challenges associated with implementing collaborative teaching in vascular surgery training programs in developing countries.

By addressing these objectives, we aim to provide evidence on the feasibility and effectiveness of collaborative teaching as a strategy to improve the training and capabilities of vascular surgeons in resource-limited settings.

## 2 Methods

### 2.1 Study aim and objectives

The aim of this study is to evaluate the effectiveness of collaborative teaching among ultrasonographers, anesthesiologists, and vascular surgeons in the specialty training of vascular surgeons in developing countries. The objectives are to assess improvements in ultrasonography and basic anesthesia skills among trainees, measure their overall satisfaction, and identify the benefits and challenges of implementing this teaching approach.

### 2.2 Research design

This study employed a mixed-methods approach with a pre-test post-test quasi-experimental design, without a control group. The design was chosen to measure the impact of the collaborative teaching intervention on trainees’ skills and satisfaction levels.

### 2.3 Participants

The participants were vascular surgery trainees from developing country, affiliated with the Department of Vascular Surgery at Sichuan Provincial People’s Hospital in Chengdu, China.

Inclusion criteria were:

1. Enrollment in a vascular surgery training program.
2. Willingness to participate in the study and provide informed consent.

Exclusion criteria included:

1. Previous extensive training in ultrasonography or anesthesia.
2. Inability to attend the entire training program.

A total sample population of 30 trainees was recruited, with 30 completing the study.

### 2.4 Recruitment methods

Participants were recruited through announcements at medical institutions. Interested trainees completed a screening questionnaire to confirm eligibility.

### 2.5 Pre-test post-test quasi-experimental design

Participants underwent pre-test and post-test assessments to evaluate their baseline skills in ultrasonography and anesthesia and to measure any improvements following the intervention. The assessment process involved both theoretical exams and practical operations tailored specifically for the trainees’ level of knowledge and experience in vascular surgery.

#### 2.5.1 Skill assessment tests

##### 2.5.1.1 Ultrasound skills assessment

The ultrasound skills assessment was designed to evaluate trainees’ proficiency in basic ultrasound operation techniques and image interpretation relevant to vascular surgery. The test included both a written component, covering ultrasound principles and vascular anatomy, and a practical component, where trainees were asked to perform ultrasound scans on standardized phantoms or volunteer patients under supervision. A standardized scoring system was developed based on the ability to correctly identify anatomical structures, manipulate the ultrasound machine, and interpret images accurately. This scoring system was reviewed and validated by a panel of experienced ultrasonographers prior to the study.

##### 2.5.1.2 Anesthesia skills assessment

The anesthesia skills assessment focused on trainees’ knowledge and proficiency in basic anesthesia techniques, particularly nerve block anesthesia relevant to vascular surgeries. Similar to the ultrasound assessment, this test comprised both a written exam covering anesthesia principles, local anesthetic agents, and procedural steps, as well as a practical component where trainees performed simulated nerve blocks under the supervision of anesthesiologists. The scoring system was also standardized and validated by a team of anesthesiologists experienced in teaching and assessing anesthesia skills.

These assessments were designed to be challenging but achievable for trainees with varying levels of experience, ensuring that improvements in skills could be accurately measured over the course of the intervention. The lack of a control group is acknowledged as a limitation affecting internal validity but was necessary due to practical constraints.

### 2.6 Data collection tools

Data collection involved tests, questionnaires, and interviews. These instruments were developed by the research team and validated through pilot testing. The tests assessed practical skills, while the questionnaires gathered demographic information and satisfaction levels. Interviews provided qualitative insights into the trainees’ experiences.

#### 2.6.1 Examples of data collection tools

1. Skill assessment test: A practical test to measure proficiency in ultrasonography and basic anesthesia techniques.
2. Satisfaction questionnaire: A Likert-scale questionnaire assessing trainees’ satisfaction with the collaborative teaching approach.
3. Interview guide: A semi-structured guide for conducting qualitative interviews with participants. We conducted in-depth interviews with the participating ultrasonographers, anesthesiologists, and vascular surgeons. The interview content primarily focused on their views on collaborative teaching, experience sharing, and suggestions for future teaching. Through these interviews, we aimed to gain a deeper understanding of the actual operation and potential issues of collaborative teaching, providing a basis for subsequent improvement and optimization.

### 2.7 Inter-rater reliability and baseline assessments

To ensure inter-rater reliability, multiple assessors were trained to use standardized criteria for evaluating skills. Baseline assessments were conducted by experienced faculty members using predefined criteria for ultrasonography and anesthesia skills.

### 2.8 Curriculum delivery timetable

An example timetable for the curriculum delivery was as follows:

Week 1–2: Intensive workshops on ultrasonography and anesthesia basics.

Week 3–4: Hands-on practice sessions under supervision.

Week 5–6: Case studies and collaborative problem-solving exercises.

### 2.9 Teaching implementation and case presentation

During the teaching implementation phase, we designed a systematic collaborative teaching curriculum. The curriculum content includes but is not limited to ultrasound operation techniques, image interpretation, diagnostic thinking, nerve blocks, and local anesthesia skills. We utilized various teaching methods such as case analysis, hands-on practice and group discussions, and simulated surgeries to enhance the trainees’ practical operation abilities and interdisciplinary collaboration skills.

During the teaching process, the ultrasonographers were responsible for teaching ultrasound operation techniques and image interpretation skills to ensure that trainees could master the usage of ultrasound equipment and image interpretation techniques. The anesthesiologists taught nerve block and local anesthesia techniques to help trainees grasp the key points of anesthesia management during surgery. The vascular surgeons integrated ultrasound and anesthesia knowledge, guiding trainees in practical operations and surgical simulations to improve their clinical practice abilities and interdisciplinary collaboration skills.

#### 2.9.1 Case presentation

Taking lower extremity varicose veins, the most common surgical disease in vascular surgery, as an example. During high ligation and stripping surgery of the great saphenous vein, femoral nerve block anesthesia can be performed under ultrasound guidance, thus replacing general anesthesia or spinal anesthesia. This enables the surgeon to complete the surgery independently. The specific implementation process and effects of this training model can be described in detail as follows. Firstly, the ultrasonographer uses ultrasound equipment to locate the target femoral nerve, explains the regional structures in the images, and enables learners to clearly understand the specific meanings of the ultrasound images, identifying the femoral nerve, femoral arteriovenous, muscle tissue, and possible variations. Then, under the guidance of the ultrasonographer, the anesthesiologist performs the nerve block, explaining the ratio of local anesthetics, dosage, drug delivery techniques, and methods for managing possible complications. Finally, the vascular surgeon instructor performs high ligation and stripping surgery on the great saphenous vein, explaining and guiding the entire process. This collaborative teaching model allows learners to learn and master ultrasound-guided femoral nerve block techniques through practical operations. They can then independently complete high ligation and stripping surgery of the great saphenous vein without the involvement of ultrasonographers and anesthesiologists, which is particularly significant for hospitals in rural areas or developing countries lacking equipment and medical personnel.

### 2.10 Analysis methods and software

Quantitative data were analyzed using GraphPad Prism software, employing statistical tests such as paired t-tests to compare pre- and post-test scores. For the interview content, we conducted thematic analysis, summarizing and extracting the interviewees’ viewpoints and suggestions to identify the characteristics, advantages, and potential issues and improvement directions of collaborative teaching.

## 3 Results

### 3.1 Participant demographics

A total of 30 trainees were recruited for this study, including 6 vascular surgery interns, 18 resident physicians, and 6 specialist doctors. The average age of the trainees was 27.3 years old, with males accounting for 83.3% and females accounting for 16.7%. Their professional experience in vascular surgery ranged from 0 to 10 years.

### 3.2 Improvement in trainees’ skills

By comparing the skill test data of trainees before and after teaching, we found significant improvements in their ultrasound operation skills and basic anesthesia skills. The following Table 1 details the skill improvements of the trainees:

**Table 1:** Students’ skill improvement status.

Skill	Pre-teaching skill score (*N* = 30)	Post-teaching skill score (*N* = 30)	Skill improvement score (*N* = 30)	R square	*p*
Ultrasound skills	39.03 ± 2.093	72.67 ± 1.674	33.63 ± 2.680	0.8516	<0.0001
Anesthesia skills	39.03 ± 1.782	73.00 ± 1.165	33.97 ± 2.129	0.9299	<0.0001

For the ultrasound and anesthesia skills assessments, we specifically designed scoring systems that took into account the training objectives and the knowledge and experience levels of the trainees. These systems aim to comprehensively evaluate the proficiency of trainees in their respective skills and ensure consistency in scoring across all participants.

#### 3.2.1 Ultrasound skills assessment scoring system

##### 3.2.1.1 Written component (50% of total score)

This part assesses trainees’ knowledge of ultrasound principles, vascular anatomy, and basic ultrasound machine operation. It consists of multiple-choice and single-choice questions. Each correct answer is awarded a fixed number of points, with a maximum of 50 points achievable.

##### 3.2.1.2 Practical component (50% of total score)

The practical component evaluates trainees’ ability to correctly identify anatomical structures, manipulate the ultrasound machine, and interpret images accurately. The assessment is conducted on volunteer patients under supervision. The scoring criteria include:

1. Accurate identification of anatomical structures (e.g., vessels, muscles, nerves).
2. Proper machine settings and manipulation (e.g., depth, gain adjustments).
3. Clear and accurate image interpretation.
4. Time taken to complete the task.

A standardized checklist is used to score the practical component, with a maximum of 50 points achievable.

#### 3.2.2 Anesthesia skills assessment scoring system

##### 3.2.2.1 Written component (50% of total score)

Similar to the ultrasound assessment, this part evaluates trainees’ knowledge of anesthesia principles, local anesthetic agents, procedural steps, and possible complications. It comprises multiple-choice and single-choice questions, with a maximum of 50 points achievable.

##### 3.2.2.2 Practical component (50% of total score)

The practical assessment focuses on trainees’ proficiency in performing simulated nerve blocks under ultrasound guidance. The assessment criteria encompass:

1. Correct selection and preparation of equipment and medications.
2. Accurate localization of the target nerve under ultrasound guidance.
3. Appropriate injection technique and local anesthetic spread.
4. Ability to manage potential complications.

A standardized checklist is used to score the practical component, with a maximum of 50 points achievable.

Prior to the study, both scoring systems were reviewed and validated by panels of experienced ultrasonographers and anesthesiologists, respectively, to ensure their reliability and validity.

The statistical analysis results of skill improvement among different professional experience groups (Table 2) indicate that: 1. the ultrasound skills and basic anesthesia skills of each professional experience group have been significantly improved after the training, and 2. ANOVA results show that there is a significant difference in the improvement of ultrasound skills between the Interns group and the Fellow Doctors group (*p* = 0.001). This may be related to their lower skill levels before the training, which provided them with greater potential for improvement. There is no significant difference in the improvement of basic anesthesia skills among the groups (*p* = 0.256).

**Table 2:** Statistical analysis results of skill improvement among different professional experience groups.

Analysis item	Group	Mean improvement in ultrasound skills	Mean improvement in basic anesthesia skills	Significant difference (*p*-value)
Descriptive statistics	Interns group	50.00	35.00	–
	Residents group	30.50	36.00	–
	Fellow doctors group	22.50	30.00	–
ANOVA	Improvement in ultrasound skills	–	–	0.001* (Interns vs. fellow doctors)
	improvement in basic anesthesia skills	–	–	0.256 (No significant difference)

### 3.3 Trainee satisfaction evaluation

The results of the questionnaire survey revealed that trainees were satisfied with the collaborative teaching model. The following Table 3 summarizes the trainees’ satisfaction evaluation of collaborative teaching:

**Table 3:** Students’ satisfaction evaluation of collaborative teaching.

Satisfaction evaluation	Number of respondents	Percentage
Very satisfied	20	67%
Satisfied	8	27%
Neutral	1	3%
Dissatisfied	1	3%

### 3.4 Characteristics and advantages of collaborative teaching

Through thematic analysis of the in-depth interviews with the participating ultrasonographers, anesthesiologists, and vascular surgeons, we have identified the following characteristics and advantages of collaborative teaching:

1. Interdisciplinary cooperation: Collaborative teaching integrates knowledge and skills from multiple disciplines such as ultrasound, anesthesia, and vascular surgery, enabling interdisciplinary cooperation and exchange.
2. Strong practical orientation: The teaching process emphasizes hands-on practice and simulated surgeries, allowing trainees to experience and learn firsthand, thus enhancing their clinical practice abilities.
3. Positive feedback from trainees: Trainees generally express that the collaborative teaching model helps improve their professional skills and interdisciplinary collaboration abilities, and they are satisfied with the teaching effect.

These characteristics and advantages demonstrate the unique role and value of collaborative teaching in training vascular surgeons in developing countries. In the future, we can further promote and optimize this teaching model to enhance the quality and effectiveness of vascular surgeon training.

## 4 Discussion

This study delved into the unique role and value of collaborative teaching in training vascular surgeons in developing countries through its implementation. In the results section, we presented significant improvements in trainees’ ultrasound operation skills and basic anesthesia skills, along with their satisfaction evaluations of collaborative teaching. These data provide compelling evidence supporting the effectiveness of the collaborative teaching model in vascular surgeon training.

Collaborative teaching has been increasingly recognized as an effective educational approach in various healthcare disciplines (13). Recent studies have demonstrated its potential to enhance learning outcomes, improve interprofessional collaboration, and optimize the use of limited resources, particularly in developing low- and middle-income countries. The World Health Organization (WHO) has emphasized the importance of task shifting and collaborative approaches to address the shortage of specialized healthcare professionals in low- and middle-income countries (11, 14).

Task shifting involves reallocating tasks from highly trained health workers to those with less training but sufficient skills. This strategy has been shown to enhance workforce efficiency and improve healthcare delivery (15, 16). For example, a study by Callaghan, Ford, and Schneider systematically reviewed task-shifting initiatives for HIV treatment in Africa and found significant improvements in patient outcomes and healthcare accessibility (17). Similarly, Mullan and Frehywot highlighted the effectiveness of non-physician clinicians in sub-Saharan Africa in addressing the healthcare workforce crisis (18).

In the context of vascular surgery training, integrating ultrasonographers, anesthesiologists, and vascular surgeons through collaborative teaching can significantly enhance trainees’ skills and knowledge. Previous studies have shown that interprofessional education (IPE) can improve communication, teamwork, and clinical competencies among healthcare trainees (19, 20). However, the application of collaborative teaching in vascular surgery training remains underexplored. Our study aims to fill this gap by evaluating the impact of a collaborative teaching model on the skills and satisfaction of vascular surgery trainees in developing countries.

The critical appraisal of the literature indicates that while task shifting and collaborative approaches are beneficial, their implementation requires careful planning and support. Challenges such as resistance to change, the need for standardized training, and ensuring the quality of care must be addressed (10, 16).

Our study contributes to the growing body of evidence supporting collaborative teaching in healthcare education. It provides insights into its feasibility and effectiveness in resource-limited settings and offers recommendations for future research and practice.

Firstly, the collaborative teaching model integrates knowledge and skills from multiple disciplines such as ultrasound, anesthesia, and vascular surgery through interdisciplinary cooperation and exchange. This integration not only enables trainees to comprehensively grasp relevant knowledge in the field of vascular surgery but also provides them with opportunities to collaborate with doctors from different professional backgrounds. In clinical practice in vascular surgery, interdisciplinary cooperation is crucial. For example, during complex vascular surgeries, ultrasonographers can provide precise vascular images and blood flow information, anesthesiologists ensure the stability of patients’ vital signs, and vascular surgeons formulate surgical plans and perform surgeries based on this information. Through collaborative teaching, trainees can develop a sense of interdisciplinary collaboration during their learning process, laying a solid foundation for their future clinical practice.

Secondly, the collaborative teaching model emphasizes hands-on practice, allowing trainees to experience and learn firsthand. This teaching method is more engaging than traditional theoretical lectures, stimulating trainees’ interest and enthusiasm for learning (21). Through practical operations, trainees can gain a deeper understanding of theoretical knowledge, master operational skills, and enhance their clinical practice abilities (22).

Moreover, trainees’ satisfaction evaluations of collaborative teaching indicate that this teaching model meets their learning needs and improves their professional skills and interdisciplinary collaboration abilities. Trainees generally express that through collaborative teaching, they have not only acquired more knowledge and skills but also learned how to collaborate with doctors from other professional backgrounds, enhancing the efficiency and quality of clinical work. The positive feedback on this teaching model provides valuable experience and insights for our future teaching work.

Finally, the collaborative teaching model is particularly significant for developing countries, where access to advanced medical equipment and professional technology is often limited (10, 16). By training vascular surgeons in ultrasound techniques, image interpretation, and anesthesia skills, this approach equips them with essential tools and skills to provide more comprehensive and effective treatment for patients under limited equipment and human resources. Additionally, promoting interdisciplinary collaboration enables trainees to effectively work with multidisciplinary teams, improving the overall quality of treatment in these countries (23).

However, this study also has some limitations. Firstly, due to time and resource constraints, the number of trainees recruited was limited, which may affect the generalizability of the results. Future studies can expand the sample size to further validate the effectiveness of the collaborative teaching model. Secondly, this study primarily employed qualitative research methods, which can deeply explore the characteristics and advantages of collaborative teaching but lack quantitative data support. Future studies can combine quantitative research methods to collect more data for analysis and validation.

## 5 Conclusion

The collaborative teaching model plays a unique role and value in training vascular surgeons in developing countries. Through interdisciplinary cooperation and exchange, emphasis on hands-on practice and simulated surgeries, and positive feedback from trainees, this teaching model can comprehensively improve trainees’ professional skills and interdisciplinary collaboration abilities. In the future, we can further promote and optimize this teaching model to cultivate more outstanding vascular surgical talents in developing countries, providing patients with higher-quality and more efficient medical services. Simultaneously, we need to pay attention to the limitations and deficiencies of the study, continuously improving and refining research methods and approaches to promote the continuous development and progress of medical education.

To maximize the benefits and overcome the challenges associated with this teaching model, the following recommendations are proposed: Medical institutions should provide strong support for the implementation of collaborative teaching models, including administrative backing and resource allocation. Developing standardized training protocols and guidelines can ensure consistency and uniformity in the delivery of training across different sessions and instructors. Regular assessment and feedback mechanisms should be established to monitor the progress of trainees and the effectiveness of the training program, allowing for timely adjustments and improvements. Training programs for faculty members should be conducted to familiarize them with the collaborative teaching model and address any resistance to change. This can help in creating a supportive and conducive learning environment. Organizing interdisciplinary workshops and seminars can further enhance communication, teamwork, and mutual understanding among different specialties, fostering a culture of collaboration. Finally, the collaborative teaching model should be designed to be scalable and adaptable to different contexts and settings, allowing for broader implementation and impact.

## Data Availability

The raw data supporting the conclusions of this article will be made available by the authors, without undue reservation.
